# Size matters: the impact of nucleus size on results from spatial transcriptomics

**DOI:** 10.1186/s12967-023-04129-z

**Published:** 2023-04-21

**Authors:** Elyas Mohammadi, Katarzyna Chojnowska, Michał Bieńkowski, Anna Kostecka, Magdalena Koczkowska, Michał A. Żmijewski, Marcin Jąkalski, Martin Ingelsson, Natalia Filipowicz, Paweł Olszewski, Hanna Davies, Justyna M. Wierzbicka, Bradley T. Hyman, Jan P. Dumanski, Arkadiusz Piotrowski, Jakub Mieczkowski

**Affiliations:** 1 3P-Medicine Laboratory, Medical University of Gdańsk, 80 210 Gdańsk, Poland; 2 Department of Pathomorphology, Medical University of Gdańsk, 80 210 Gdańsk, Poland; 3 Department of Histology, Medical University of Gdańsk, 80 210 Gdańsk, Poland; 4 Department of Public Health and Caring Sciences, Geriatrics, Rudbeck Laboratory, Uppsala University, 751 85 Uppsala, Sweden; 5 Krembil Brain Institute, University Health Network, Toronto, ON M5G 2C4 Canada; 6 Department of Medicine and Tanz Centre for Research in Neurodegenerative Diseases, University of Toronto, Toronto, ON M5S 1A8 Canada; 7 Department of Immunology, Genetics and Pathology and Science for Life Laboratory, Uppsala University, 751 85 Uppsala, Sweden; 8 Department of Neurology, Massachusetts General Hospital, Boston, MA 02114 USA; 9 Massachusetts Alzheimer’s Disease Research Center, Charlestown, MA 02129 USA; 10 Harvard Medical School, Boston, MA 02115 USA

**Keywords:** Spatial transcriptomics, Cerebral cortex, Neuronal nuclei, Consecutive tissue sections, Data integration

## Abstract

**Background:**

Visium Spatial Gene Expression (ST) is a method combining histological spatial information with transcriptomics profiles directly from tissue sections. The use of spatial information has made it possible to discover new modes of gene expression regulations. However, in the ST experiment, the nucleus size of cells may exceed the thickness of a tissue slice. This may, in turn, negatively affect comprehensive capturing the transcriptomics profile in a single slice, especially for tissues having large differences in the size of nuclei.

**Methods:**

Here, we defined the effect of Consecutive Slices Data Integration (CSDI) on unveiling accurate spot clustering and deconvolution of spatial transcriptomic spots in human postmortem brains. By considering the histological information as reference, we assessed the improvement of unsupervised clustering and single nuclei RNA-seq and ST data integration before and after CSDI.

**Results:**

Apart from the escalated number of defined clusters representing neuronal layers, the pattern of clusters in consecutive sections was concordant only after CSDI. Besides, the assigned cell labels to spots matches the histological pattern of tissue sections after CSDI.

**Conclusion:**

CSDI can be applied to investigate consecutive sections studied with ST in the human cerebral cortex, avoiding misinterpretation of spot clustering and annotation, increasing accuracy of cell recognition as well as improvement in uncovering the layers of grey matter in the human brain.

**Supplementary Information:**

The online version contains supplementary material available at 10.1186/s12967-023-04129-z.

## Background

The spatial transcriptomics concept has been introduced as a combination of massively parallel sequencing and microscopic imaging [1]. This method is an attractive approach in studies of normal development and in clinical translational research. Visium Spatial Gene Expression (ST) is one of the technologies developed around this concept. ST is a next-generation molecular profiling method dedicated to unraveling the transcriptomic architecture of the tissue. The application of ST for mapping the transcriptome with the morphological context has been proven successful in many fields [2].

Although ST is a powerful new technique for capturing patterns of spatial distribution of gene expression, it also has a drawback of its design. A Visium Gene Expression slide consists of two or four tissue-capture areas (6.5 mm × 6.5 mm), divided into 4992 spots, each 55 µm in diameter. Every spot contains oligonucleotide probes with unique sequence barcodes that encode spatial information in gene expression data (Asp et al., 2020). Due to their size, spots may encompass the expression profiles of several cells. Consequently, this diminishes the accuracy of distinguishing neighboring cell types. This can be addressed by several methods [3, 4], including integration with other single-cell analyses [5]. The most popular methods for the integration rely on so called anchors, which represent similar gene expression patterns.

The importance of the anchor-based data integration in distinct single-cell modalities (i.e., spatial transcriptomics and single nucleus RNA sequencing data [snRNA-seq]) has been investigated previously [5]. However, the application of Consecutive Slices Data Integration (CSDI) in ST analysis using the anchor-based approach remains unexplored. We investigated the effects of CSDI on spot clustering and cell-type annotation using both snRNA-seq and ST technologies in human cerebral cortex samples. By applying the CSDI to ST, we aimed to evaluate whether a single slice of tissue would be sufficient for ST analysis or whether consecutive slices would be required. We found that without CSDI, the pattern of obtained spot clusters between consecutive slices is inconsistent, and the cell-type annotation does not match the microscopic characterisation of the slice. These issues were resolved by employing CSDI, and layer-structure of grey matter of the human brain was unveiled.

## Methods

### Data acquisition

We utilized the modified 10 × Genomics Visium Spatial Gene Expression method to analyze the profiles of consecutive sections from fresh-frozen brain tissues. Accordingly, we used the orbitofrontal (ON) and temporal neocortex (TN) samples from two subjects. Tissue specimens were provided by Harvard University and Massachusetts Alzheimer’s Disease Research Center and all experimental procedures were conducted in accordance with Independent Bioethics Committee for Scientific Research at Medical University of Gdansk (consent No. NKBBN/564-108/2022). The brain-tissue slices were placed onto a Visium Gene Expression slide (10x Genomics) and fixed according to the 10x Genomics protocol (doc. CG000239 Rev. C). Next, the slides were divided into two via a piece of silicone gasket. Subsequently, we stained the tissue by two methods—hematoxylin and eosin, and hematoxylin and Congo red—to detect eventual amyloid deposits. We imaged the slides at 20× magnification using brightfield settings (Olympus cellSens Dimension software). Afterward, the tissue was permeabilized, using conditions according to manufacturer protocol. The mRNA was released and bound to spatially barcoded capture probes on the slide. Next, cDNA was synthesized from captured mRNA, and sequencing libraries were prepared. Samples were loaded and pooled according to the protocol (doc. CG000239 Rev. C) and sequenced in the standard Illumina pair-end constructs, using Illumina’s NextSeq 550 System.

### Visium data processing

Four pairs (consecutive slices) of ST raw data (BCL files) from two postmortem brain samples were converted to fastq files using 10x Genomics software Space Ranger version 1.2.1 and its *spaceranger mkfastq* function. Subsequently, reads were aligned to the human genome-reference sequence (GRCH38) using the STAR method, and spatial feature counts were generated using the *spaceranger count* function. Because an inverted microscope was used for imaging, all images were flipped horizontally prior to being applied to the Space Ranger*.*

### Data preprocessing and normalization

All outputs from *spaceranger count* were read as Seurat objects using *Load10X_Spatial* function of Seurat version 4.0.3. Prior to data normalization, the percentages of mitochondrial genes were calculated by the *PercentageFeatureSet* function. Then, the spots with a number of spatial features of more than 7000 and less than 200 were removed; spots that encompassed more than 15% of mitochondrial genes also were omitted from the downstream analyses. Standard normalization was performed using the *NormalizeData* function and the *LogNormalize* method using default parameters. Variable features for each object were determined using the *FindVariableFeatures* function and *VST* method. Next, the data were scaled and regressed out for the percentage of mitochondrial genes using the *ScaleData* function.

### Dimensionality reduction and clustering

Dimensionality reduction was completed using the *RunPCA* function. Prior to clustering, nearest neighbors were determined by the *FindNeighbors* function with default parameters. After this, the *FindClusters* function determined the clusters by a shared nearest-neighbor (SNN) modularity optimization-based clustering algorithm (The resolution was arbitrarily set to 0.3).

### Consecutive slices data integration

Dimensionality reduction for consecutive slices was completed jointly through diagonalized canonical correlation analysis (CCA). Using the *FindIntegrationAnchors* function, mutual nearest neighbors (MNNs) were found in this shared low-dimensional space and were termed anchors (for more details, see Stuart et al. [5]). The *IntegrateData* function was considered for CSDI using precomputed anchor sets. The integrated consecutive slices were saved as transcriptomics-corrected objects. The same workflow for dimensionality reduction and clustering was applied to integrated objects. Finally, the eight Seurat objects (four pairs of consecutive slices) before and after CSDI (16 in total) were saved as RDS files to be compared from different perspectives.

### Label transferring from snRNA-seq to ST

The previously annotated snRNA-seq dataset was obtained from scREAD, a publicly available snRNA-seq database [6] (https://bmbls.bmi.osumc.edu/scread/). The causes of death for the two donors were Alzheimer’s disease (Subject A) and stroke (Subject B). Hence, snRNA-seq profiles with AD01104 and AD01102 scREAD data IDs for Alzheimer’s and non-Alzheimer’s disease were retrieved [7]. The raw-sequencing data and the digital-expression matrices obtained using the 10x Genomics software Cell Ranger are available in the NCBI’s Gene Expression Omnibus (GSE129308) and are accessible through the GEO Series accession number GSM3704357-GSM3704375 (Otero-Garcia et al., 2020).

Data normalization and dimensionality reduction with the same parameters as the ST data were conducted for the two snRNA-seq datasets. By considering snRNA-seq as our reference and ST data as query datasets, anchors were found, and precomputed cell labels were transferred using the *FindTransferAnchors* (identifying shared cell/spot states present across different datasets) and *TransferData* functions, respectively. Label transferring and clustering were completed twice for each of the ST objects—once before and once after CSDI—to investigate the effect of CSDI on label transferring and clustering.

## Results

**Fig. 1:**
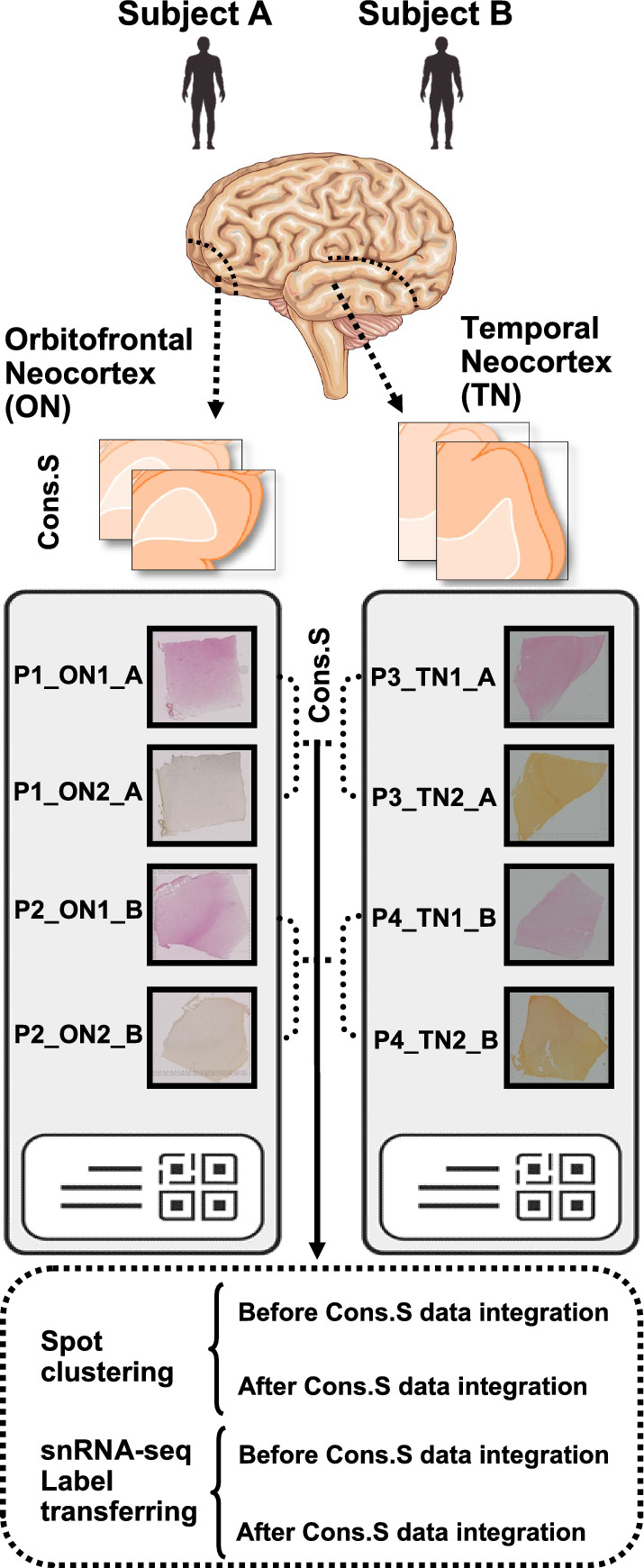
Schematic view of experimental workflow and in silico analytical pipeline. Four pairs (P1–P4) of consecutive slices of human postmortem brains were obtained from two distinct anatomical locations (ON and TN). Two types of computational analyses were performed (spot clustering and snRNA-seq label transferring) and further divided into additional subtypes (before and after CSDI for each category). The names of slices are as follows: P, pairs of consecutive-sections; ON (1–2), Orbitofrontal Neocortex (section number in pair of consecutive slices), TN (1–2): Temporal Neocortex (section number in pair of consecutive slices); A and B: two studied subjects; Cons. S: consecutive sections

We conducted spatial gene expression analysis in human postmortem, fresh frozen tissue sections. Two anatomical regions, the Orbitofrontal Neocortex (ON) and the Temporal Neocortex (TN) from two adult male donors were investigated (Fig. 1). From each region of both subjects, one pair of consecutive slices (eight slices in total) were prepared. We cut the ON and TN tissue into 10–12 µm thick sections. Each sample was sequenced to a median depth of 187 million reads, corresponding to a mean of 3300 unique molecular identifiers (UMIs) and a mean of 2058 genes per spot.

### Identifying distinct cell types and their annotation using single tissue section

**Fig. 2:**
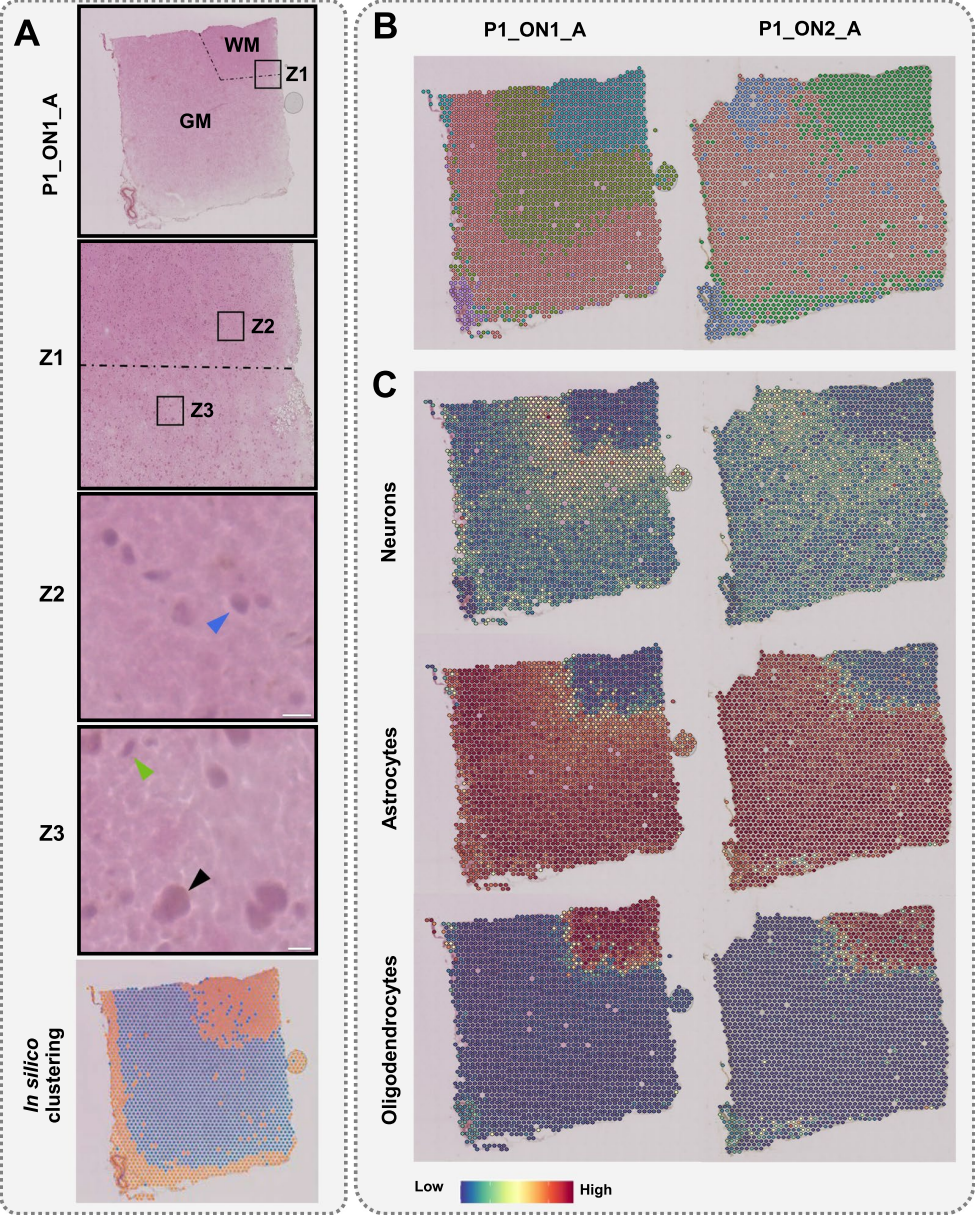
Results from spatial transcriptomics analysis using single tissue sections. **A** Top, histological image of orbitofrontal neocortex (ON) with marked white matter (WM) and grey matter (GM); Z1, zoomed-in image of the border between WM and GM; Z2, Blue arrow points to an oligodendrocyte nucleus; Z3, Black and Green arrows represent nuclei of neurons and astrocytes, respectively. In Z2 and Z3, white scale bars represent 10 µm. Bottom, unsupervised classification of spots. **B** The ST spots clustering before CSDI. **C** Label transferring before CSDI. The name of the sample encodes number of the section (P1/P2), number of slice (ON1/ON2), and patient id (A/B)

Figure 2A shows the distinction between the grey matter (GM) and the white matter (WM). The border between GM and WM was established histologically based on cellular composition and arrangement (Fig. 2A, Z1, Z2, and Z3). We used an unsupervised method to investigate whether categorizing the ST spots based on their transcriptomics profile could represent structural layers of the brain. Subsequently, we compared the obtained groups with histological images of tissue slices to assess the obtained clusterization and classification (Fig. 2A). Thus, we confirmed the general consistency of GM and WM patterns revealed by histologic and transcriptomic methods.

We performed spot clustering using the steps recommended by Satija et al. [7] in order to cluster the spots with similar expression profiles, and distinguish distinct cellular layers. The resulting clusters revealed the separation of subcortical WM and cortical GM. More detailed morphological layers of the brain were also unveiled through the more detailed clustering (Fig. 2B). Considering the expected similarity of architecture between two consecutive slices of the cerebral cortex, we should observe the very similar pattern of clusters. However, this consistency was vague, and the layered structure of GM in P1_ON2_A could not be observed (Fig. 2B). We observed that although, the use of a single section of brain tissue with the ST method can be informative, it may also have critical limitations in spot clustering. To overcome this, we decided to use external gene expression data set and anchor-based integration method.

To better understand the identified brain layers, we integrated the measured expression profiles with a previously described snRNA-seq dataset [8]. As single nucleus profiles contain greater number of genes than in our ST profiles, the integration of these two data sets allowed us to perform the spot annotation more precisely. Using predefined cell-type annotations in snRNA-seq—including oligodendrocytes, astrocytes, and neurons—the ST spots were labeled (see “Methods” for details). The pattern of transferred labels is shown in P1_ON1_A and P1_ON2_A as an example (Fig. 2C). The locations of oligodendrocytes and astrocytes were primarily identified in WM and GM, respectively, in line with brain structure (Fig. 2A). However, we could not confidently annotate neurons in GM, which is incompatible with histology (Fig. 2A). In summary, a single slice of brain tissue using the ST method is informative but has limitations in distinguishing cell types using label transferring as well as in spot clustering.

### The effects of CSDI on identifying distinct cell types and their annotations

Stuart et al. [5] developed the CSDI to correct the transcriptomic profiles of consecutive slices using anchors representing spots with similar gene expression profile from two consecutive slices. This is used to pair spots from the two slices. At the same time, the transcriptomic differences between pairs of spots in anchors are used to correct datasets from both consecutive sections.

**Fig. 3:**
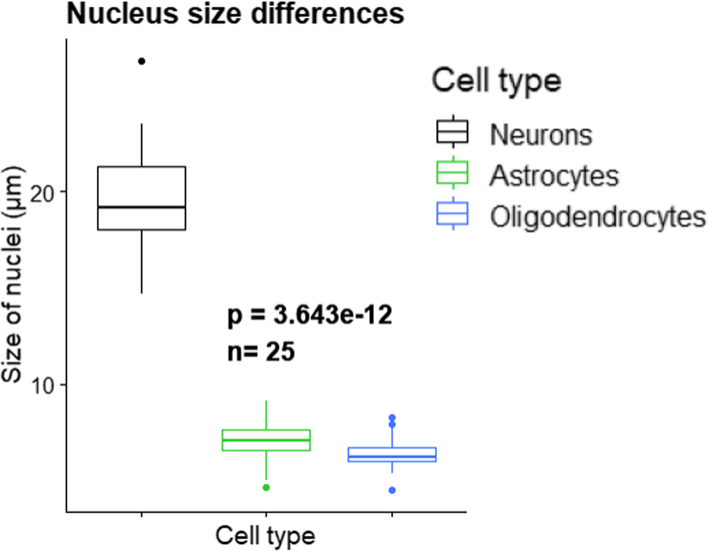
The nucleus size heterogeneity among investigated cell types. The boxplot shows significant differences in size of nuclei in the human cerebral cortex (Kruskal–Wallis test). This is compatible with histological differences of nuclei size in Fig. 2A, Z2 and Z3 which the scale bars denote 10 µm

**Fig. 4:**
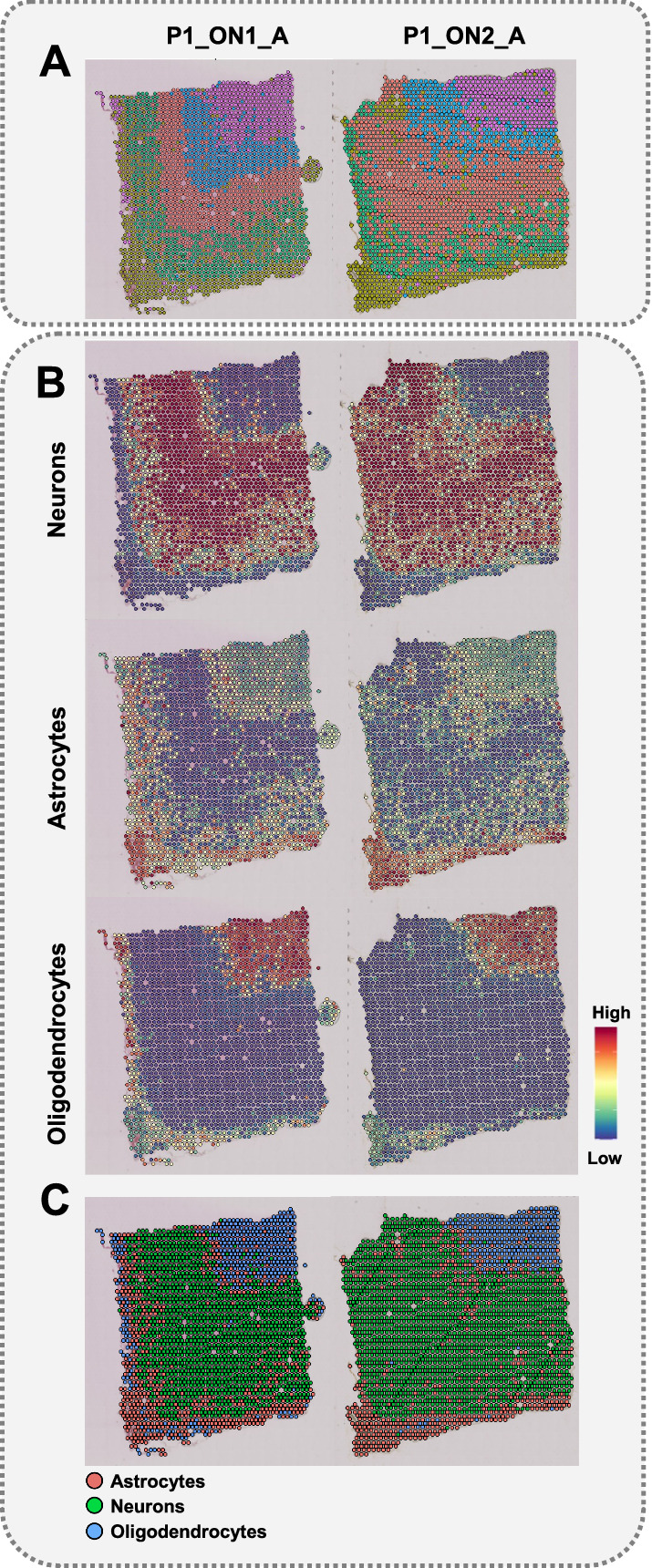
Improvement of the spot clustering and annotation using CSDI method. **A** Clustering after CSDI unveiled the GM layers and resolved the inconsistency between the pattern of clusters in consecutive slices. **B** snRNA-seq Label transferring after CSDI. The annotation probability is shown as a scheme for three different cell types. **C** The classification of spots through label transferring is shown by considering the highest probability of annotation for each spot after CSDI

We decided to apply the CSDI method due to the heterogeneity of the brain in terms of size of nuclei among different cell types (Fig. 3). On average, the size of a nucleus from a neuron in GM (about 20 μm) is much larger than the thickness of tissue section (10–12 μm). Consequently, a single tissue section will encompass only part of nuclei for essentially all neurons present in the studied sample. This restriction will also apply to other smaller nuclei, although to a lesser extent. Thus, for all cells present in a studied brain tissue, it will cause partial loss of transcriptomic signals. Taking “P1_ON1_A” and “P1_ON2_A” as consecutive slices of ON as an example, we could identify all the morphological layers of the brain [9] only after CSDI (Fig. 4A and Additional file 1: Fig. S1). In conclusion, CSDI can resolve the issue of inconsistency of the pattern of clustering between consecutive slices. Moreover, by applying the same parameters (see “Methods” for details), we can identify more neuronal layers in GM [10] (Fig. 4A and Additional file 1: Fig. S1).

Cell bodies of neurons are mainly found in the GM (Fig. 2A). However, during our label transferring, the spots marked as neurons received weak probability values in the GM (Fig. 2C). This is an important concern, which led us to hypothesize that using information from a single section of tissue may lead to inaccurate interpretation of clusters and cell types. Our approach to transferring cell labels from snRNA-seq to ST before and after CSDI revealed different results, which provides support for the above hypothesis. These differences are much more pronounced in GM, where the spots recognized as neurons, or astrocytes are the dominating cell types (Fig. 4B). Accordingly, we compared the annotations with consideration for the size of nuclei and the structural layers of the brain (WM and GM). Prior to CSDI, the annotation of neurons and astrocytes received low and high probabilities, respectively, in the area of the GM, while the nuclei of oligodendrocytes were mostly visible in the WM (Fig. 2C and Additional file 2: Fig. S2). Interestingly, after CSDI, the likelihood of the annotation of neurons was increased due to the gain of neuronal transcriptomic profiles (Fig. 4B), which is consistent with the histological imaging (Fig. 2A). It is noteworthy that we did not observe any changes in the probability of annotation for oligodendrocytes in the WM before and after CSDI, which is also in agreement with histological structure of the cerebral cortex. Among the transferred labels from snRNA-seq to ST, neurons and astrocytes are mainly available in GM. Consequently, no signal fluctuation will occur before and after CSDI in WM. In both situations, the WM would preferentially be annotated with oligodendrocytes (Figs. 2C, 4B, and Additional file 2: Fig. S2).

The differences between annotations obtained for the spots before and after CSDI can be attributed to the fact that the size of neuronal nuclei is much bigger than astrocytic nuclei [11] (Fig. 3). Their size is actually larger than the thicknesses of the tissue sections used in the ST protocol. Accordingly, a single slice will capture incomplete transcriptomic neuronal context. CSDI provides a robust means of rectification of this misinterpretation. Hence, the corrected signals of all types of nuclei can be obtained. Consequently, the label transferring from snRNA-seq to ST is made consistent with the histological findings (Fig. 2A). Ultimately, one can study the spatial distribution of different cell types more precisely.

We evaluated the results from two independent spot-categorization methods used in this study: label transferring and spot clustering. Hence, we classified the spots using the transferred labels (Fig. 4C and Additional file 3: Fig. S3) and compared them with the spot clustering results represented in Fig. 4A. We observed that the green cluster in Fig. 4C represents the three distinguished neuronal layers in Fig. 4A (blue, red, and green clusters). We were not capable of labeling the neuronal layers in Fig. 4C as the utilized reference snRNAseq dataset did not distinguish neuronal subtypes. Similarly, the blue cluster in Fig. 4C represents oligodendrocytes in Fig. 4A (purple cluster). Through determining the locations of neurons and oligodendrocytes in both methods, we demonstrated that the results from both spot-categorization methods are consistent with the histological images (Fig. 2A).

**Fig. 5:**
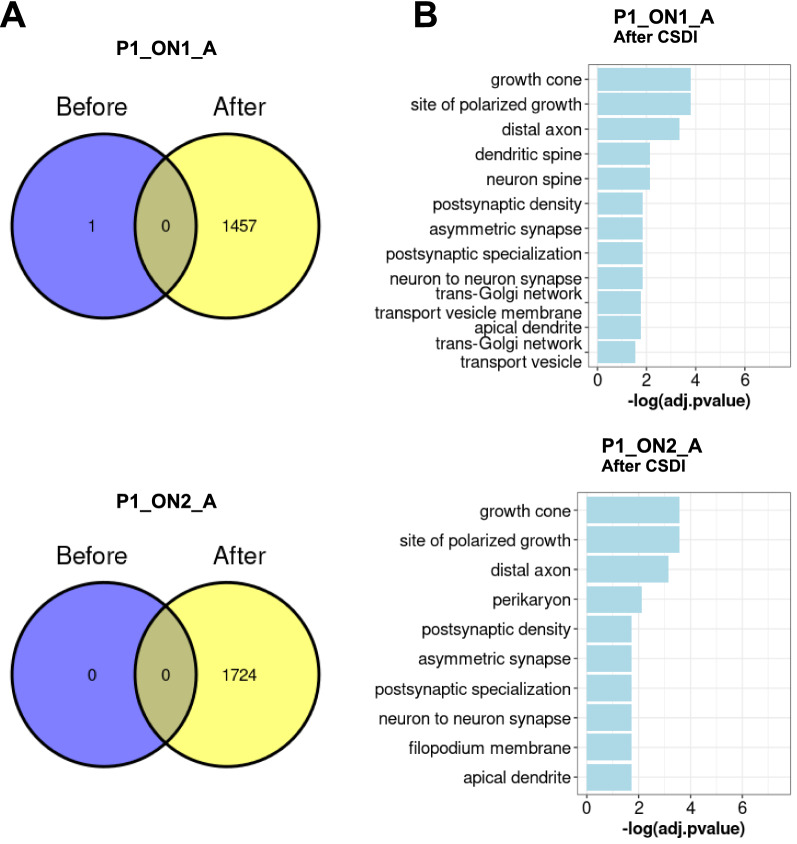
Improvement of the results from DEGs in spots assigned to neurons, before and after CSDI. **A** Intersection of the spots assigned to neurons before (purple circle) and after (yellow circle) CSDI. **B** Gene ontology analysis is applied to DEGs in neurons versus oligodendrocytes and astrocytes

We assessed the improvement of annotation for neurons before and after CSDI. A considerable rise in the number of defined neurons was observed after data integration (Fig. 5A and Additional file 4: Fig. S4). To investigate the accuracy of changes in spot labeling, we computed the differentially expressed genes (DEGs) in neurons versus oligodendrocytes and astrocytes before (if available) and after CSDI. In some of the tissue sections, no neurons could be identified before CSDI (Fig. 5 and Additional file 4: Fig. S4). Next, we applied the DEGs to Gene Ontology (GO) analysis (Fig. 5B and Additional file 4: Fig. S4). According to the enriched terms in GO analysis, neurons were identified accurately after data integration. We showed that by applying CSDI, mislabeled neuronal spots will be rectified. Thus, more accurate biologically meaningful results can be achieved.

## Discussion

We studied the impact of the CSDI method on spatial gene expression analysis and evaluated the effect of CSDI on the improvement of clustering and label transferring [5]. The application of CSDI was motivated by the two issues we observed in the results of the basic spatial transcriptomic analysis. Firstly, in the GM, we observed inconsistencies between the patterns of clusters in consecutive slices (Fig. 4A). Secondly, we failed to recreate the expected layered structure of GM (Fig. 4A). According to the study conducted by Maynard et al. [10], data correction in consecutive slices was performed using the data-refinement step of Space Ranger. Hence, the spatial topography of gene expression in the human dorsolateral prefrontal cortex was defined. The pattern of determining clusters was consistent in all pairs of consecutive slices, a phenomenon we observed only after applying the CSDI. Moreover, using CSDI, we distinguished cortical and subcortical WM layers. Thus, we showed that the expected consistency of the pattern of clustering between consecutive slices can be achieved with CSDI similar to Space Ranger. We investigated the results of the clustering and label transferring, with and without CSDI utilization. Simultaneously, we compared the consistency of the results obtained with the topographic organization of the cerebral cortex. We observed the improvement of clustering and the label transferring after applying CSDI. The superior performance of using CSDI is likely related to the size of nuclei in different cell types as the determining parameter. The sizes of the nuclei of certain neurons are much larger than the nuclei of astrocytes and oligodendrocytes (e.g., neurons from the pyramidal layer (Figs. 2A and 3) of the cerebral cortex) [11]. In the human brain, the size of neuronal nuclei may exceed the tissue thickness recommended in the cryosectioning step of the ST protocol (10 µm) [12]. This may jeopardize capturing the whole transcriptomics profile using a single slice.

High resolution spatial methods [13] and/or experiments involving tissue sections or entire organ cross-sections from small animals are virtually free from the risk of losing the transcriptomics content of cells. Akeret et al. [14], studied single tissue sections of mice brains using 10 × Visium spatial transcriptomics without any problem in spot labeling. The reason could be due to the fact that in mice, the average diameter of neuronal soma derived from the cortical pyramidal layer does not exceed 10 µm [15]. Hence, our approach is specifically applicable to tissues composed of cells with nuclei sizes exceeding the minimum thickness of the section required for the 10x Genomics Visium spatial transcriptomics experiment.

We used a combination of ST and snRNA-seq technologies to unveil the cerebral-cortex structure and related cell types. The ST preserves the spatial location of gene expression. However, its resolution at the level of the spot, as well as in terms of the number of captured genes, is nominally lower than the single-nuclei/single-cell transcriptomics [16]. The lower resolution results from the size of spots in ST expression slides (55 µm in diameter). Accordingly, each spot may encompass the transcriptomic profiles of multiple cells. The ST data integration with snRNA-seq/scRNA-seq is considered a deconvolution method to unravel the underlying cell types in each ST spot. In this context, using snRNA-seq has advantages over scRNA-seq. This is because the process of tissue cryopreservation ruptures the cell membranes; however, nuclear membranes remain intact during the freeze–thaw cycle [17]. Furthermore, it has been shown that the RNA-seq of single nuclei is highly representative of transcriptional profiles from the entire cells. This fact is specifically relevant to postmortem brain samples after long-term storage at − 80 °C [17]. Hence, we utilized the prelabeled snRNA-seq to deconvolute the ST spots.

To confirm the deconvolution of ST spots and defined cell types, we compared our annotation with neuropathological findings. Astrocytes play a vital role in delivering energy to neurons via the astrocyte-neuron lactate shuttle [18]. Hence, astrocytic nuclei are spatially located beside perikarya (Fig. 2A), mostly placed in the GM [19]. In Fig. 4B, the GM is annotated for both neurons and astrocytes, corresponding to the previous findings [18]. According to the shape of oligodendrocytic nuclei—which are round with visible halos [20]—the annotation of WM for oligodendrocytes corresponds with the expected normal morphology of the brain cross-section [21] (Fig. 2A). These concepts are consistent with our histological (WM and GM) (Fig. 2A) and cell-type (astrocytes, neurons, and oligodendrocytes) (Fig. 4C) classifications.

An alternative solution to resolve the low resolution of the ST method is to decrease the size of barcoded spots in gene expression slide glasses. However, as we addressed in our study, ST results would be affected by the size of neuronal nuclei because the origin of the problem is not the sizes of spots but the thickness of the tissue slices. Accordingly, by decreasing the sizes of capture spots, deconvolution methods may no longer be required anymore; however, the need for CSDI remains.

In summary, the transcriptomic profiles of ST consecutive slices may need to be corrected prior to further analysis. Correcting the datasets simply for the depth of sequencing using normalization methods (e.g., log normalization) cannot remove all the unknown batch effects of consecutive slices. Data correction can be performed during the data-processing step by Space Ranger using the *spaceranger aggr* function or during the analysis steps using CSDI. In Space Ranger, the transcriptomic profile of consecutive slices will be aggregated, normalized to the same sequencing depth. Then, the feature-barcode matrices and the analysis of the combined data can be recomputed. In CSDI, the spots with similar transcriptomics profiles in two datasets will be anchored. Using the anchors, the transcriptomics profile of consecutive slices will be corrected, and one can proceed with the downstream analysis. Consequently, the results of clustering and annotation will be improved after data correction. Therefore, more trustable biological findings can be achieved. A general comparison between CSDI and *Space Ranger aggr* is shown in Additional file 4: Fig. S4. The pattern of clusters after applying CSDI is more consistent in consecutive slices than Space Ranger (Additional file 5: Fig. S5, A), while in label transferring both methods perform equally (Additional file 5: Fig. S5, B). However, more in depth analysis is required to show the outperformance of one over the other.

The study has potential limitations. First, while spatial transcriptomic technology allowed us to define the spatial location of cell types in human brain tissues, the resolution was limited to 1–10 cells per spot [22]. This means that spatial transcriptomic analysis should be taken with caution and possibly benefit from the computational approaches like CSDI. In this study, to annotate the ST spots more precisely, we integrated snRNA-seq and ST profiles. Second, we could only examine the role of neuronal nucleus size from human brain on results from ST. Although, we acknowledged that this is not a concern in mouse brains [14, 15], we do not have enough relevant information about other species. In addition, we exclusively used the 10 µm thickness for the tissue sections of the human brain, as recommended by manufacturer. However, a wider range of tissue thicknesses could provide a more comprehensive understanding of how neuronal nucleus size may affect the ST analysis.

## Conclusion

We observed that the thickness of tissue sections may be an important factor of the spatial transcriptomic analysis. In particular, the recommended in the cryosectioning step of the ST protocol (10 µm) [12] may not be sufficient to capture the entire transcriptomics profile of human brain tissue due to the large size of neuronal nuclei (about 20 μm). Importantly, this limitation can be overcome by using CSDI, which adjust the transcriptomic profiles prior to further analysis. The amendment leads to improved annotation results and more reliable biological findings.

### Limitations of the study

Despite that ST is a powerful technology, the current study has the following limitations: (1) we couldn’t attribute the gene changes to a particular cell type among multiple cell types captured in spots, therefore we need to use deconvolution methods to decipher the spots content; (2) the size of neuronal nuclei exceed the recommended thickness of tissue slices, thus the transcriptomics profile of spots encompassing neurons might be misinterpreted (3) the size of slide’s capture area did not allow us to study any anatomic region completely.

## Supplementary Information

**Additional file 1: Figure S1.** Improvement of the spot clustering through CSDI. The colors represent the results of unsupervised clustering performed either before **(A)** or after **(B)** application of CSDI. **(A)** The expected consistency of the pattern of spot clusters between consecutive slices is missed. **(B)** The consistency of clustering after consecutive slices data integration is improved. The patients IDs are marked above the pictures. The designations “before” and “after” refer to pictures made either before or after CSDI respectively.

**Additional file 2: Figure S2.** Improvement of the label transferring by CSDI. The annotation probability is shown as a scheme for three different cell types. Label transferring from snRNA-seq to ST consecutive slices is shown before and after data integration. Before Integration: the probability of spot annotations for neurons is not compatible with tissue histology. After integration: the probability of the presence of neurons increased in the GM of the cerebral cortex.

**Additional file 3: Figure S3.** A comparison between the pattern of spot classification after CSDI through spot clustering and label transferring methods. The consistency between the results from these two methods, which is compatible with the histological information of tissue slices, supports the accuracy of the spot categorization.

**Additional file 4: Figure S4.** Improvement of the results from DEGs in spots assigned to neurons, before and after CSDI. Venn diagrams represent the intersection of spots assigned to neurons before (purple circle) and after (yellow circle) CSDI. In both situations, DEGs in neurons versus oligodendrocytes and astrocytes (if available) were applied to GO analysis and the results are shown in barplots.

**Additional file 5: Figure S5.** A comparison between CSDI and Space Ranger aggr in improvement of spot clustering and label transferring. Using CSDI, A) the pattern of clusters are more consistent versus Space Ranger aggr while in B) label transferring both methods perform equally.

## Data Availability

The snRNA-seq data used in this study are publicly available at the scREAD database (https://bmbls.bmi.osumc.edu/scread/) under AD01104 and AD01102 scREAD data IDs. Additionally, the snRNA-seq raw data and the digital-expression matrices obtained using the 10 × Genomics software Cell Ranger are available in the NCBI’s Gene Expression Omnibus (GSE129308) and are accessible through the GEO series accession numbers GSM3704357-GSM3704375. The spatial transcriptomics data used in this study are privately available at the GEO data repository under the GSE184510 accession number. These data can be available from the authors upon a reasonable request.

## Abbreviations

ST: Spatial gene expression
CSDI: Consecutive slices data integration
snRNA-seq: Single nucleus RNA sequencing
ON: Orbitofrontal neocortex
TN: Temporal neocortex
SNN: Shared nearest-neighbor
CCA: Canonical correlation analysis
MNNs: Mutual nearest neighbors
DEGs: Differentially expressed genes
GO: Gene Ontology

## Code availability

The scripts used in this study are developed by the R programming language version 4.1.0 and have been deposited in a public Github repository (https://github.com/jakubmie/ST_snRNA-seq).
