# Supplementary material for: Size matters: the impact of nucleus size on results from spatial transcriptomics
Source: J Transl Med. 2023 Apr 21;21:270. doi: 10.1186/s12967-023-04129-z (PMC10120157; doi:10.1186/s12967-023-04129-z)
Supplement: Supplementary file 2 — Additional file 2: Figure S2. Improvement of the label transferring by CSDI. The annotation probability is shown as a scheme for three different cell types. Label transferring from snRNA-seq to ST consecutive slices is shown before and after data integration. Before Integration: the probability of spot annotations for neurons is not compatible with tissue histology. After integration: the probability of the presence of neurons increased in the GM of the cerebral cortex. [file 12967_2023_4129_MOESM2_ESM.pdf]

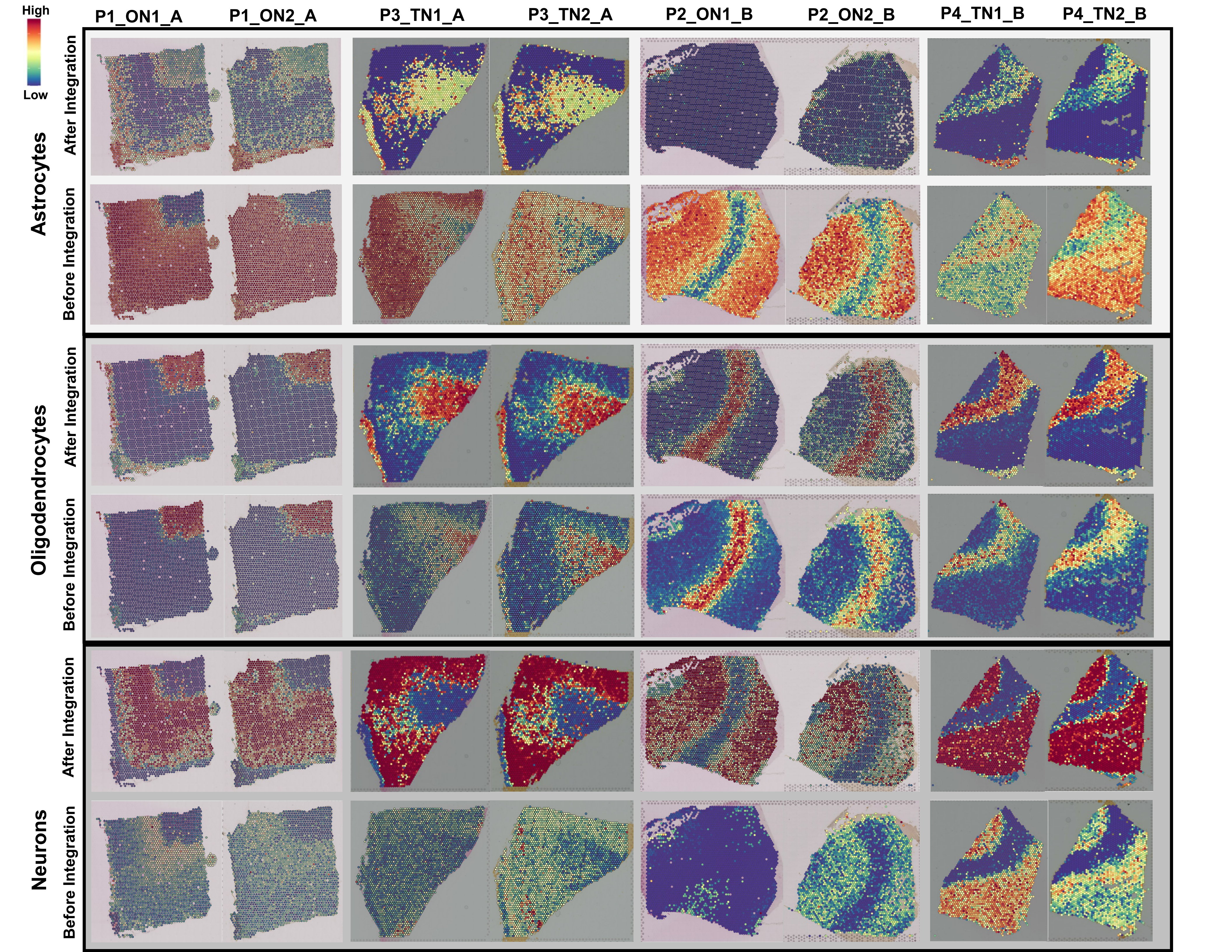
High
Low
P1_ON1_A
P1_ON2_A
P3_TN1_A
P3_TN2_A
P2_ON1_B
P2_ON2_B
P4_TN1_B
P4_TN2_B
Astrocytes
After Integration
Before Integration
Oligodendrocytes
After Integration
Before Integration
Neurons
After Integration
Before Integration
