# Supplementary material for: Size matters: the impact of nucleus size on results from spatial transcriptomics
Source: J Transl Med. 2023 Apr 21;21:270. doi: 10.1186/s12967-023-04129-z (PMC10120157; doi:10.1186/s12967-023-04129-z)
Supplement: Supplementary file 3 — Additional file 3: Figure S3. A comparison between the pattern of spot classification after CSDI through spot clustering and label transferring methods. The consistency between the results from these two methods, which is compatible with the histological information of tissue slices, supports the accuracy of the spot categorization. [file 12967_2023_4129_MOESM3_ESM.pdf]

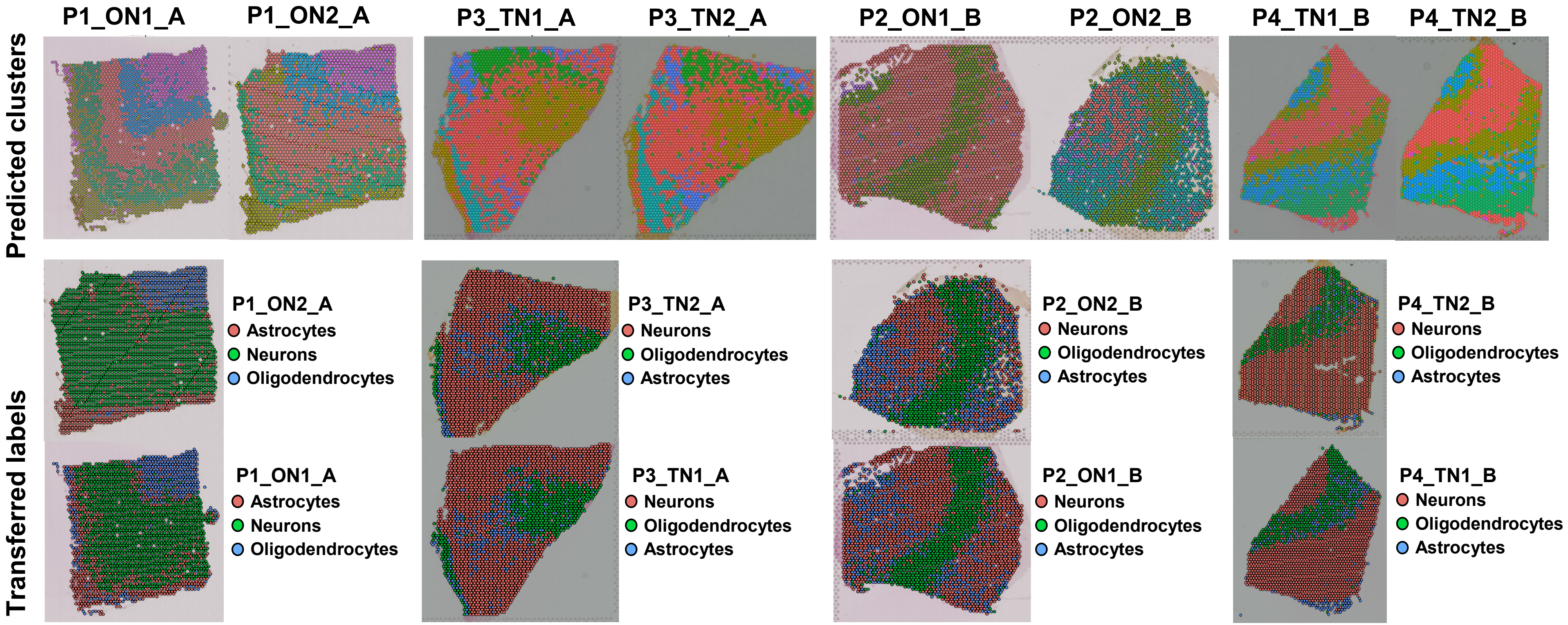

Predicted clusters
Transferred labels
P1_ON1_A
P1_ON2_A
P3_TN1_A
P3_TN2_A
P2_ON1_B
P2_ON2_B
P4_TN1_B
P4_TN2_B
P1_ON2_A
Astrocytes
Neurons
Oligodendrocytes
P1_ON1_A
Astrocytes
Neurons
Oligodendrocytes
P3_TN2_A
Neurons
Oligodendrocytes
Astrocytes
P3_TN1_A
Neurons
Oligodendrocytes
Astrocytes
P2_ON2_B
Neurons
Oligodendrocytes
Astrocytes
P2_ON1_B
Neurons
Oligodendrocytes
Astrocytes
P4_TN2_B
Neurons
Oligodendrocytes
Astrocytes
P4_TN1_B
Neurons
Oligodendrocytes
Astrocytes
