# Supplementary material for: Size matters: the impact of nucleus size on results from spatial transcriptomics
Source: J Transl Med. 2023 Apr 21;21:270. doi: 10.1186/s12967-023-04129-z (PMC10120157; doi:10.1186/s12967-023-04129-z)

GO for the upregulated genes in spots assigned to neurons before and after integration

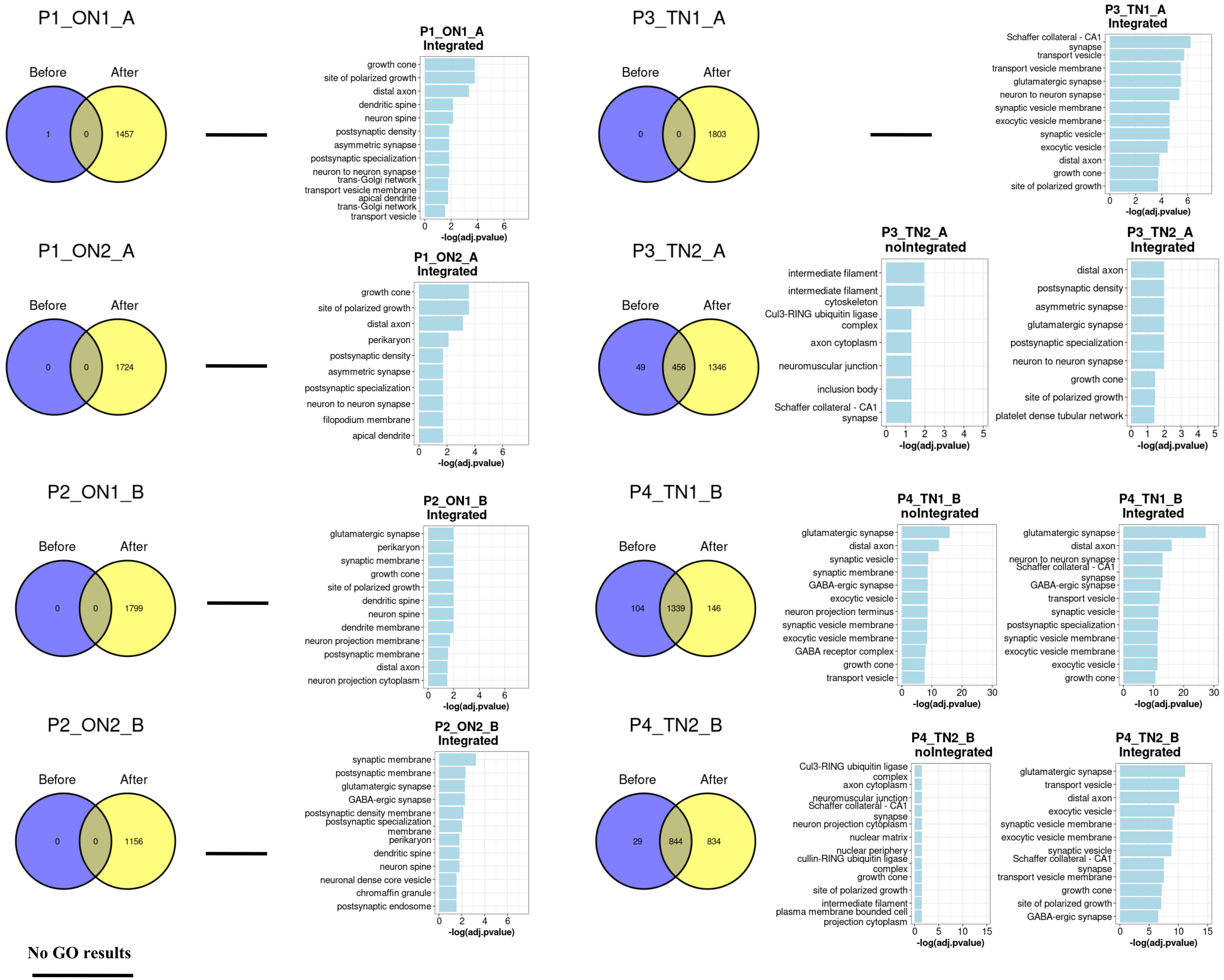

Supplement: Supplementary file 4 — Additional file 4: Figure S4. Improvement of the results from DEGs in spots assigned to neurons, before and after CSDI. Venn diagrams represent the intersection of spots assigned to neurons before (purple circle) and after (yellow circle) CSDI. In both situations, DEGs in neurons versus oligodendrocytes and astrocytes (if available) were applied to GO analysis and the results are shown in barplots. [file 12967_2023_4129_MOESM4_ESM.pdf]
