# Supplementary material for: Size matters: the impact of nucleus size on results from spatial transcriptomics
Source: J Transl Med. 2023 Apr 21;21:270. doi: 10.1186/s12967-023-04129-z (PMC10120157; doi:10.1186/s12967-023-04129-z)
Supplement: Supplementary file 5 — Additional file 5: Figure S5. A comparison between CSDI and Space Ranger aggr in improvement of spot clustering and label transferring. Using CSDI, A) the pattern of clusters are more consistent versus Space Ranger aggr while in B) label transferring both methods perform equally. [file 12967_2023_4129_MOESM5_ESM.pdf]

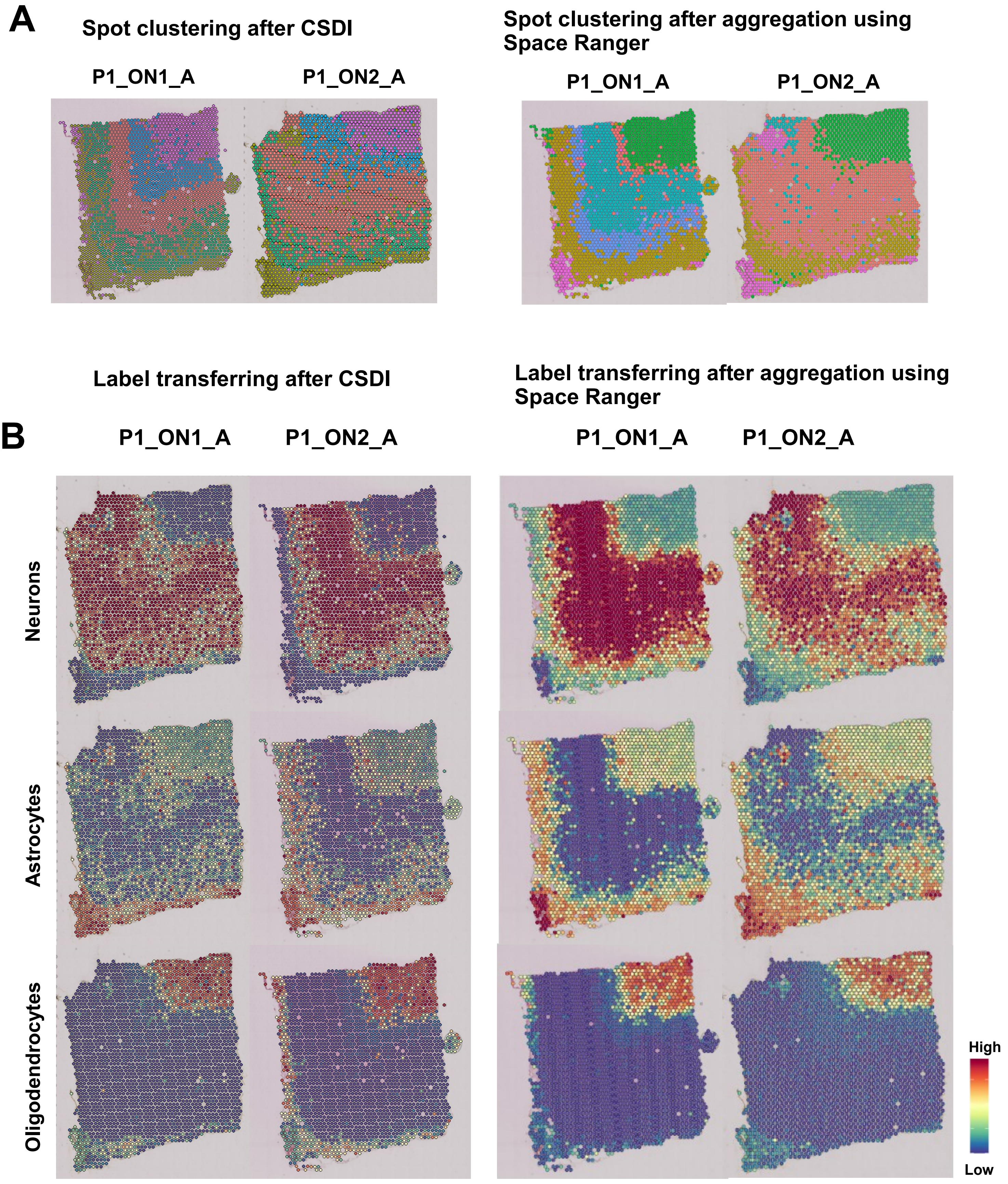
A
Spot clustering after CSDI
P1_ON1_A
P1_ON2_A
Spot clustering after aggregation using Space Ranger
P1_ON1_A
P1_ON2_A
Label transferring after CSDI
Label transferring after aggregation using Space Ranger
B
P1_ON1_A
P1_ON2_A
P1_ON1_A
P1_ON2_A
Neurons
Astrocytes
Oligodendrocytes
High
Low
