# Supplementary material for: Size matters: the impact of nucleus size on results from spatial transcriptomics
Source: J Transl Med. 2023 Apr 21;21:270. doi: 10.1186/s12967-023-04129-z (PMC10120157; doi:10.1186/s12967-023-04129-z)
Supplement: Supplementary file 1 — Additional file 1: Figure S1. Improvement of the spot clustering through CSDI. The colors represent the results of unsupervised clustering performed either before (A) or after (B) application of CSDI. (A) The expected consistency of the pattern of spot clusters between consecutive slices is missed. (B) The consistency of clustering after consecutive slices data integration is improved. The patients IDs are marked above the pictures. The designations “before” and “after” refer to pictures made either before or after CSDI respectively. [file 12967_2023_4129_MOESM1_ESM.pdf]

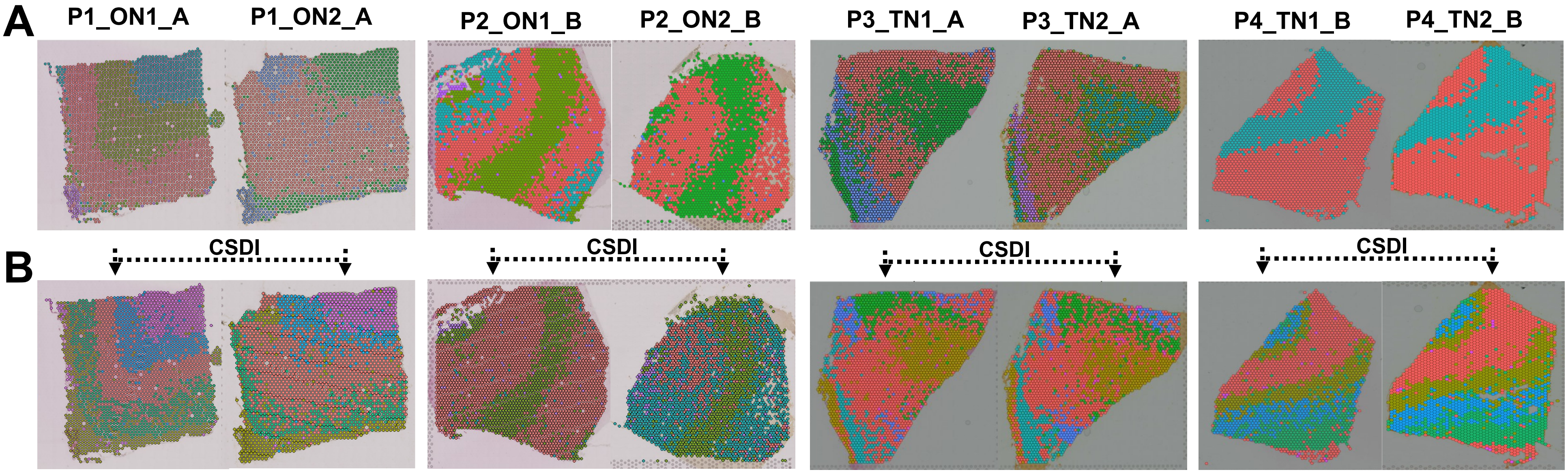

A
P1_ON1_A
P1_ON2_A
P2_ON1_B
P2_ON2_B
P3_TN1_A
P3_TN2_A
P4_TN1_B
P4_TN2_B
B
CSDI
CSDI
CSDI
CSDI
